# Preserving Organizational Resilience, Patient Safety, and Staff Retention during COVID-19 Requires a Holistic Consideration of the Psychological Safety of Healthcare Workers

**DOI:** 10.3390/ijerph17124267

**Published:** 2020-06-15

**Authors:** Pavani Rangachari, Jacquelynn L. Woods

**Affiliations:** 1 Department of Interdisciplinary Health Sciences, College of Allied Health Sciences, Augusta University, Augusta, GA 30912, USA; 2 Interventional Radiology/Trauma ICU, Wellstar Kennestone Hospital, Marietta, GA 30060, USA; jlwoods22@yahoo.com

**Keywords:** COVID-19, hospital intensive care, worker psychological safety, mental health, organizational resilience, patient safety, staff retention, leadership

## Abstract

During the COVID-19 pandemic, healthcare workers are fighting a lethal virus with acute shortages of Personal Protective Equipment (PPE). These unprecedented circumstances have amplified the sources of emotional distress and worker burnout. However, many healthcare organizations (HCOs) in the United States, have opted for a “stoic approach” to healthcare worker support, i.e., no additional support beyond federal and state policy protections for the licensing and liability of healthcare workers. In this scenario, a key public health concern is sustaining an adequate healthcare workforce, both by way of quantity (adequate numbers) and quality (maximizing clinician resilience to provide safe care to large volumes of patients under challenging conditions). Therefore, it is imperative for HCO leaders to recognize that a limited view of worker psychological safety, without due consideration for the broader emotional distress created by the pandemic, could have the effect of restricting organizational resilience and adversely impacting patient safety and staff retention during and beyond the pandemic. This paper uses the organizational resilience framework to discuss the potential impact of a stoic approach to healthcare worker support on patient safety and staff retention in a hospital intensive care unit (ICU) during COVID-19. The discussion in turn, helps to develop recommendations for HCOs to overcome these challenges.

## 1. Introduction

During normal circumstances, working in healthcare is recognized to be emotionally distressing [1]. With the arrival of COVID-19, healthcare workers are fighting a lethal virus with PPE shortages and no evidence-based treatment. These unprecedented conditions have greatly amplified the sources of emotional distress experienced by healthcare workers [2,3,4]. Without adequate PPE protection, the paramount fear expressed by healthcare workers is that they will not only get sick, but also spread the virus to their patients and families. As such, many have opted to socially isolate themselves within their own homes [5]. Concurrently, healthcare workers are being forced to handle life and death situations on the frontlines, like never before. While patient care decisions were historically based on patient preferences, during COVID-19, with limited resources, these decisions are being based on triaging protocols, creating moral distress among healthcare workers, as they are being called upon to triage patients knowing there are a limited number of ICU beds and ventilators [6,7]. Other sources of emotional distress include extreme workloads, rapidly evolving practice environments (e.g., non-ICU nurses serving in makeshift ICUs), and witnessing large volumes of medication errors, infections, and deaths [4,5,6,7,8,9,10,11,12,13,14,15].

Under these unprecedented circumstances, the USA has witnessed a surge in federal and state legislation in support of healthcare workers, including looser professional licensing, credentialing, and point-of-care restrictions, limits and immunities to healthcare provider liability, deployment of medical students, use of “volunteer” or retired practitioners, repurposing of units and beds never intended for intensive care, and “sharing” of ventilators [16,17,18,19]. The common theme therefore, has been expanding capacity to enable healthcare providers to handle extreme caseloads. Cumulatively, such legislation has had the effect of enhancing job security for healthcare workers, in areas most affected by the pandemic.

However, within this context, many hospitals and healthcare organizations (HCOs) in the United States, have opted for a “stoic approach” to healthcare worker support, i.e., no additional support beyond the federal and state policy protections, for the broader emotional distress and risks endured by healthcare workers during the pandemic [6,20,21,22,23,24]. For example, the dire shortages of PPE during early days of pandemic, prompted the USA Centers for Disease Control & Prevention (CDC) to issue directives to healthcare workers to improvise with materials at hand, to develop face masks. In this context, hospital leaders are reported to have encouraged healthcare workers to use homemade masks for protection, as if nothing had changed, i.e., with no acknowledgment for the lower protection offered by homemade masks, compared to surgical masks and N-95 masks [20,21]. Similarly, when encountered with nurses feeling overwhelmed and anxious of making a mistake while treating an unfamiliar patient base, hospital managers are reported to have made comments such as “everyone is out of their comfort zone, hang in there”, or “we hear your concerns, but there’s nothing we can do” [6,21,22]. In another instance, hospital leaders are reported to have gone around ICU nursing stations handing out wipes for frontline workers to use before wearing masks (so they are reusable), without asking how staff was doing, or if they needed anything [21]. Lack of visibility of hospital leaders on the frontlines of care, has also been reported to be a serious concern [21,22].

In summary, a growing body of pandemic literature has reported concern in regard to lack of reassurance, support, and acknowledgment from HCO leaders, for the unprecedented level of emotional distress experienced by frontline healthcare workers during COVID-19 [6,7,8,9,10,11,12,13,14,15,20,21,22,23,24]. Under normal circumstances, healthcare workers could seek solace from workplace stress with family and social lives. This no longer remains an option during COVID-19. As such, worker burnout from emotional distress has become a growing concern during the pandemic [2,3,4]. In this scenario, a key public health concern being reported in the pandemic literature, is sustaining an adequate healthcare workforce, both by way of quantity (adequate numbers of healthcare workers), and quality (maximizing clinician resilience to provide safe and effective care to large volumes of patients under challenging conditions) [12,13,14,15].

### Purpose of This Paper

A key concern of HCO (hospital) leaders during COVID-19, is their organization’s ability to be resilient in adapting to the rapidly evolving pressures of the pandemic, to provide safe and effective care to their patients and communities [25,26]. Given this primary concern, it is imperative for HCO leaders to recognize that a limited view of worker psychological safety (solely in terms of job security), without due consideration for the broader emotional distress created by the pandemic, could have the effect of severely restricting organizational resilience and adversely impacting patient safety and staff retention during and beyond the pandemic. The purpose of this paper is to use the organizational resilience framework to discuss the potential impact of a stoic approach to healthcare worker support on resilience, patient safety and staff retention within a hospital ICU context, during the COVID-19 pandemic. The discussion, in turn, helps to develop recommendations for HCO leaders to overcome these challenges, ensure patient safety, and retain a resilient healthcare workforce during and beyond the pandemic period.

## 2. Framework for Organizational Resilience in HCOs

On the frontlines of healthcare, resilience has been described as the ability to improvise with materials at hand to develop solutions to unexpected problems, thereby enabling patient care to be delivered safely despite obstacles [27]. Organizational resilience is known to have three interconnected levels: (i) the individual level, for example, individual healthcare workers who use workarounds to temporarily resolve recurring safety problems on the frontlines, and then communicate their safety concerns to managers, in an effort to prevent problem recurrence; (ii) the team level, for example, managers who encourage frontline healthcare workers to freely communicate their safety concerns, with a view to addressing underlying issues and preventing problem recurrence; and (iii) the organizational level, for example, senior leadership commitment to patient safety and lasting improvement (change) [28,29]. In other words, resilience can be described as a property of individuals, teams, and the whole organization.

The literature on organizational resilience has described three key elements of resilience, including (1) foresight (ability to predict something bad could happen), (2) coping (ability to prevent something bad from becoming worse), and (3) recovery (ability to recover from a bad occurrence). Each of these elements in turn, can occur at each of the three levels outlined above (individual, team, and organization) [27,28,29,30,31]. When error recovery through workarounds remains restricted to the individual level, it is referred to as ‘first-order problem solving.’ On the face of it, first-order problem solving appears successful because it allows patient care continues in the short term, however, its downside is the lack of communication about failures which keeps managers unaware of the need for change and prevents problems from being investigated, making it likely for problems to recur [27]. First-order problem-solving can advance to ‘second-order problem solving’ when staff feel safe to speak up about safety concerns to managers who are in a position to address the underlying causes (by developing systems or experimenting with solutions). Second-order problem solving is necessary for the organization to learn from individual error recovery and ensure lasting improvement, i.e., apply safety standards for all patients, with minimal variation [27,28,29,30].

A resilient organization is one whose workers are supported in the key elements of foresight, coping, and recovery across the three levels (individual, team, and organization), so that safety is promoted at an organizational level, by anticipating failures, by learning how to adapt to circumstances of failure, and by restoring safe conditions after failure. The patient care process of clinical handover has been used to illustrate the concept of resilience in HCOs [31,32]. Clinical handover is internationally recognized as a patient safety priority since it represents a disruption to the continuity of care and so may be especially prone to errors leading to patient harm. When considering foresight, coping, and recovery elements in clinical handover across the three levels, an example of foresight at an individual level, would be a clinician contacting their replacement before shift change. At an organizational level, it would be workforce training and standard operating procedures about handover. Likewise, an example of coping at the individual level would be a clinician agreeing to stay on until a replacement is found. At an organizational level, it would be extensive documentation of clinical care by the day people identifying high-risk situations overnight. Lastly, an example of recovery at the individual level would be the clinician making the effort to ensure that supervisors are aware of the situation, as well as any patient care concerns emanating from that shift. At an organizational level, it would be reviewing the team’s management of the service and clinical practice, via an executive walk-around.

The above examples explain how foresight, coping and recovery strategies at the individual level, may involve dynamic tradeoffs initiated by healthcare workers to recover from failures in the clinical handover process [31,32]. They also help to understand that if resilience is restricted to individual level without advancing to team and organizational levels, it could leave the organization suspended in a reactive or brittle stage of resilience (as opposed to proactive or full resilience). In such a scenario, individual workers are left to deal with failures that are likely to recur, in the absence of systems for learning from individual error recovery at the organizational level, which, in turn, allows variation to persist in individual practices, creating room for error. Such a scenario, in turn, could force an organization to be easily overwhelmed even by minor disruptions, thereby restricting organizational resilience under challenging conditions [27,31,32]. On the other hand, when systems for learning from individual error recovery (problem-solving) are developed at the organizational level, it allows resilience to advance from individual to organizational levels, which, in turn, helps to prevent problem recurrence and ensure lasting improvement [27,31]. Therefore, organizational resilience emanates from systems (e.g., communication structures) developed by leaders, to learn from foresight, coping, and recovery strategies of individual workers. In the case of clinical handover therefore, advancing from individual to organizational resilience, has the potential to transform clinical handover from a source of vulnerability, to a source of patient safety, with proactive mechanisms in place to recover from errors and prevent future process failures.

### 2.1. Worker Trust and Psychological Safety are Pre-Requisites for Organizational Resilience in HCOs

As discussed earlier, healthcare workers on the frontlines often need to make trade-offs to deal with mismatches between demand and capacity, and manage competing priorities. This requires negotiation with coworkers and flexible interpretation of organizational protocols [27,33,34]. For the individual healthcare worker, this involves taking interpersonal risks, i.e., trusting coworkers and managers to work towards a shared goal, and feeling safe in flexible interpretation of protocols to provide safe patient care [33]. In a similar vein, psychological safety has been defined as individuals’ perceptions about the consequences of taking interpersonal risks in their work environment [34]. Under normal circumstances (as opposed to COVID-19), psychological safety may be viewed in terms of the ultimate fear of losing one’s job or license, if one puts oneself on the line by asking a question, reporting a mistake, or proposing a new idea. When employees trust they will be supported, they are likely to feel psychologically safe and empowered to communicate safety concerns to managers, which, in turn, enables patient safety to improve in everyday practice for all patients [27,33]. Clearly, both trust and psychological safety are essential for healthcare workers to make tradeoffs and communicate safety concerns to managers, who are in a position to address underlying causes of errors and prevent problem recurrence. The latter, in turn, is essential for resilience to advance from individual to organizational levels. Correspondingly, the literature has underscored the importance of worker trust and psychological safety in serving as pre-requisites for organizational resilience [33].

### 2.2. Need for a Holistic Consideration of Worker Psychological Safety during COVID-19 to Provide for the Broader Impact of Emotional Distress and Burnout

It is important for HCO leaders to recognize that emotional distress emanating from a broad array of fears during COVID-19 can supersede the psychological safety of having just one fear addressed, of losing one’s job or license. Therefore, while it may be reasonable to view worker psychological safety primarily from the perspective of job security during normal times, it is essential to adopt a more holistic consideration of psychological safety during unprecedented times like COVID-19, to provide for the broader impact of emotional distress and worker burnout [12,13,14,15,35,36]. This line of reasoning is echoed by a growing stream of pandemic literature calling attention to the heightened risk of worker burnout during the pandemic [2,3,4,12,13,14,15]. For example, a recent opinion article in *JAMA* discusses a strategy for supporting the emotional well-being of healthcare workers during the pandemic, to prevent burnout [12]. The article underscores the importance of listening to the specific concerns and needs of healthcare workers and responding in a meaningful way, by facilitating access to essential services to meet those needs, along with emotional and social support. The article also cautions against recycling wellness offerings of the past (e.g., teaching generic approaches to stress reduction) during the unprecedented conditions created by COVID-19 [12,13].

Worker burnout, which has been described as one of the leading concerns for patient safety, refers to the human response to chronic emotional and interpersonal stress at work, defined by exhaustion, cynicism and inefficiency [37,38,39,40,41]. A 2017 literature review on nurse burnout showed that a key factor contributing to burnout was exclusion from the decision-making process [42]. This highlights the crucial importance of work environment and leader-empowering behaviors, in preventing worker burnout [43,44,45]. According to this literature, HCOs that implement resilience-training may experience reduced burnout, increased patient safety, and increased staff retention [38,46]. However, resilience-training by itself cannot effectively address burnout, unless the leadership strives to create a work environment of mutual trust and psychological safety, to empower healthcare workers to communicate safety concerns and participate in the decision-making process. The latter, in turn, is required for resilience to progress from individual (reactive/brittle) to organizational (proactive/full) levels [43,44,45].

### 2.3. Absence of Leadership Support for Emotional Distress during COVID-19 Could Adversely Impact Organizational Resilience, Patient Safety, and Staff Retention

At the outset of the COVID-19 pandemic, Stanford Medicine (California, USA) conducted multiple listening sessions with groups of healthcare professionals (69 total), including physicians, nurses, and advanced practice providers, to explore what healthcare workers were most concerned about and what messages and behaviors they needed most from their leaders [12,13]. These discussions identified eight sources of anxiety: (1) adequate access to PPE; (2) exposure to COVID-19 at work and taking the infection home to family, (3) inadequate access to COVID-19 testing if symptoms develop since the infection could be propagated at work, (4) uncertainty about whether their organization would support their needs if they develop infection, (5) access to childcare during school closures and increased work hours, (6) support for additional needs as work hours increase (food, lodging, transportation), (7) ability to provide competent care in a new area (e.g., non-ICU nurses deployed to serve as ICU nurses), and (8) inadequate communication and information. These eight concerns in turn, were organized into six broad requests from healthcare professionals to their organization: hear-me, prepare-me, protect-me, support-me, care-for-me, and honor-me [12,13]. To summarize, the focus group sessions found that healthcare workers need unambiguous assurance that their organizations will support their well-being during COVID-19. Above all, healthcare workers needed to be able to trust their organizations and leaders to have their backs during the COVID-19 pandemic [12,13,47,48].

The above discussion provides a broad understanding of how a stoic approach to healthcare worker support, i.e., the absence of leadership support for the broader emotional distress and burnout created by COVID-19, could lead to the erosion of trust and psychological safety needed for healthcare workers to freely communicate patient safety concerns to their managers. The latter, in turn, has the potential to severely restrict organizational resilience and adversely impact patient safety and staff retention, during and beyond the pandemic period.

## 3. Application of Organizational Resilience Framework to the Hospital ICU Context

This section applies the organizational resilience framework to the hospital ICU context, to provide a more nuanced illustration of the potential impact of a stoic approach to healthcare worker support (i.e., absence of leadership support for emotional distress during COVID-19), on organizational resilience, patient safety and staff retention, during and beyond the pandemic. The application in turn, helps to develop recommendations for HCOs to overcome these challenges, ensure patient safety, and retain a resilient healthcare workforce during and beyond the pandemic.

### Potential Impact of the “Stoic Approach” on Resilience, Patient Safety, and Staff Retention in ICUs

In areas most affected by pandemic, hospital ICUs are experiencing rapid changes in practice environments to meet the pressures of increased demands for ICU beds, with limited resources, e.g., conversion of observation units into makeshift ICUs, use of “float-pool” (non-ICU) nurses to function as ICU nurses, and in some cases, placing two patients on a single ventilator, all within the context of acute shortage (limited supply) of essential PPE [6,21,22]. In this scenario, frontline ICU nurses are faced with having to implement dynamic tradeoffs and workarounds to patient care processes on a daily basis, to resolve problems and ensure patient safety under challenging conditions [22].

For example, during COVID-19, a number of ICU patients are placed on medications (such as fentanyl, propofol, and pressor drugs) that require titration (dose adjustment) on an ongoing basis, sometimes every 5, 10, or 15 mins, to ensure effective dosage and prevent side effects. Drugs requiring titration are typically administered intravenously (IV). Under normal circumstances, drug titration requires frontline nurses to enter and exit the patient’s room multiple times within the span of an hour, to adjust dosage on the patient’s IV bag, as needed. However, amidst acute PPE shortages during the COVID-19 pandemic, the higher the frequency of entering and exiting patient rooms, the greater the risk of exposure to the virus for nurses (healthcare workers), which, in turn, increases their risk of spreading the virus to other coworkers, patients, and their families. Therefore, to mitigate their own risk of exposure amidst PPE shortages, ICU nurses are faced with having to identify innovative ways to titrate IV medications as often as needed, while limiting the frequency with which they enter and exit patient rooms. In this scenario, one potential solution could be to bring all patient IV bag-stands outside the patient rooms into the ICU hallway, to enable drug titration without entering patient rooms. However, the flip side to this dynamic trade-off, is that it poses an increased risk to patient safety by making it difficult to concurrently verify two unique identifiers prior to medication changes, one for the medication and one for the patient. Under normal circumstances, both medication and patient would be verified (scanned) every time dosage is changed, to ensure that the correct medication reaches the correct patient, in accordance with international patient safety protocols for prevention of medication errors [49]. However, bringing the IV bag-stand outside the patient room makes it difficult for the patient to be concurrently verified. Additionally, since the IV bag-stands for all COVID-19 patients are placed next to each other in a common hallway, there is greater scope for confusion as to which IV bag-stand belongs to which patient, and hence, a higher risk of medication error by way of wrong medication to the wrong patient. To mitigate this patient safety risk prior to drug titration, an individual ICU nurse may call upon a nurse colleague in the ICU to double-check that the IV bag-stand being titrated, corresponds to the correct patient (which in turn, is an example of resilience on the frontlines that enables safe care to be provided in the ICU, despite obstacles during COVID-19).

However, this novel workaround developed by some ICU nurses would need to become standard protocol at the unit level to ensure safety of all patients receiving titrated medication on the ICU. For this to happen, the nurses who developed the workarounds need to be able to freely communicate their safety concerns and workarounds with their peers and managers. However, under rapidly evolving pressures created by COVID-19, a number of barriers to communication could arise due to trust issues. For example, regular ICU nurses who originally devised the workarounds may not trust float-pool (non-ICU) nurses new to the ICU to properly implement practice changes due to inadequate training. This lack of trust, in turn, may serve as a barrier to involving float-pool nurses in workarounds that have been designed to ensure patient safety amidst rapid process changes. Such a situation in turn, has the potential to increase variation in drug titration practices in the ICU, thereby increasing the opportunity for error and patient harm. 

Similarly, communication between regular ICU nurses and managers could suffer due to lack of visibility of managers on the frontlines of ICU care. In the absence of any efforts from managers and senior hospital leaders to be present on the frontlines to understand the challenges, ICU nurses may feel betrayed by lack of emotional support from the leadership, for the unprecedented risks, pressures, and moral distress experienced on the frontlines during COVID-19. This lack of trust, in turn, has potential to considerably hinder communication related to patient safety between ICU nurses and managers. In the absence of systems for learning from individual error recovery (problem-solving) at the organizational level, resilience remains reactive (brittle) and restricted to the frontlines, with no way of advancing to team and organizational levels. This prevents the safety-benefits of novel workarounds (developed by some ICU nurses) from being applied to all patients on the unit. When resilience remains restricted to the individual level, it has potential to engender practice variations on the frontlines, thereby increasing the likelihood of unsafe practices and preventable errors. 

Another example of a workaround implemented by ICU nurses to ensure patient safety during COVID-19, may be the use of written handoff sheets (to supplement verbal handoffs) during the end-of-shift clinical handover process. Owing to large volumes of high-severity patients and time constraints in the handoff process during COVID-19, regular ICU nurses may have worked to develop a template for written handoffs to supplement verbal handoffs during the shift handover process. The written handoff sheets, in turn, may be crucial for incoming nurses to gain a quick overview of patients’ medical histories (that are used to calculate patient risk scores). The risk scores, in turn, could be vital in helping nurses determine the priorities for patient care in the unit (e.g., in responding to changes in patient conditions resulting from medication changes), which, in turn, may be crucial for patient safety. However, owing to interpersonal trust issues (discussed earlier), the regular ICU nurses who devised these novel workarounds (written handoff templates), may refrain from sharing them with their peers (e.g., float-pool nurses) and their managers, thereby propagating a pattern of reactive (brittle) resilience on the frontlines that has no way of advancing to team and organizational levels. The latter in turn, has potential to increase the potential for practice variation, errors, and preventable deaths in the ICU.

As discussed earlier, being forced to witness unsafe practices, preventable errors, and large volumes of deaths on the frontlines, has potential to create chronic emotional and interpersonal stress, leading to exhaustion and cynicism, the classic symptoms of nurse burnout [4,5,6]. Burnout resulting from chronic emotional distress in turn, has potential to result in high staff turnover and low retention not only during COVID-19, but beyond the pandemic period [2,3,4,12,13,14,15]. For example, burnout may prompt healthcare workers to contemplate moving to a different HCO (e.g., ICU in a different hospital) or to a different setting of care (e.g., from the ICU to outpatient clinic practice) or to pursue an alternate career altogether, which in turn, could endanger society’s ability to sustain an adequate healthcare workforce to fulfill HCO operations in the short run, and to meet to public health needs in the longer run, should a similar pandemic recur in the future. 

In summary, a stoic approach to healthcare worker support during the pandemic has the potential to severely restrict organizational resilience needed to recover from setbacks in patient care, by eroding trust and mitigating communication from healthcare workers to managers regarding safety concerns. The latter, in turn, has the potential to adversely impact patient safety, staff retention, and HCO operations, during and beyond the pandemic period.

## 4. Discussion: Recommendations for HCO Leadership 

The above framework application suggests that in order to preserve organizational resilience during the COVID-19 pandemic, HCO leaders need to adopt a holistic consideration of worker psychological safety—one that recognizes the complex impact of emotional distress experienced by healthcare workers during the pandemic. Meaningful support for emotional distress during the pandemic, can enable healthcare workers to trust that their organization is putting its people first, which, in turn, can enable them to feel psychologically safe and empowered to speak up about safety concerns and workarounds implemented on the frontlines [12,13,33]. Since communication between healthcare workers and managers in regard to workarounds, is crucial for resilience to advance from individual to organizational levels, the first recommendation for HCO leaders would be to create a work environment characterized by trust, psychological safety, and empowerment, to enable healthcare workers to freely communicate safety concerns with their managers. 

Concurrently, since learning from individual error recovery is crucial for advancing from reactive (brittle) resilience at the frontlines to proactive (full) resilience at the organizational level, the second recommendation for HCO leaders would be to develop communication structures that enable the organization to learn from individual error recovery (problem-solving) strategies. Advancing from reactive to proactive resilience at an organizational level in turn, can help to eliminate practice variation and enable all patients to benefit from the tradeoffs and workarounds implemented on the frontlines, to keep patients safe despite obstacles. Both sets of recommendations for HCO leaders are discussed below.

### 4.1. Recommendation 1: Create an Environment of Trust, Psychological Safety, and Empowerment to Enable Individual Workers to Communicate Patient Safety Concerns to Managers

From the earlier discussion, we can infer that a key strategy for building trust during the pandemic, would be for leaders to listen to the specific concerns of healthcare workers, understand the sources of emotional distress, assure healthcare workers that their concerns have been heard, and provide targeted support to mitigate concerns to the greatest extent possible [12,13,35,36]. For example, to address specific concerns about access to PPE, spreading infection to family, and lack of access to rapid testing, HCO leaders must provide adequate PPE, rapid access to occupational health with testing, and resources to avoid spreading the infection at home, if necessary. Similarly, to address specific concerns about not being able to provide competent care to an unfamiliar patient base, HCO leaders must provide access to training to build a critical knowledge base while striving to promote teamwork, solidarity, and communication. They must acknowledge unprecedented challenges faced by healthcare workers and encourage individuals to ask for help and rely on each other for support, rather than going it alone [12,22].

In addition to providing targeted support for concerns, leaders need to be visible on the frontlines during the pandemic, more than ever. Leaders (e.g., nursing managers, department chairs, and hospital leaders) must make an effort to visit units caring for COVID-19 patients regularly, to provide reassurance [6,12,21,22]. While leaders are not expected to have all the answers, they are expected to understand and address the daily needs of frontline workers. They are also expected to provide acknowledgment for the moral distress experienced by healthcare workers and direct them to available resources for emotional support (e.g., employee assistance program). It is also important for leaders to acknowledge the day-to-day contributions of healthcare workers during the pandemic [22]. The focus-group sessions conducted by Stanford Medicine found that an overarching request of healthcare workers is be honored with simple and genuine expressions of gratitude for their willingness to put themselves in harm’s way for patients [12].

When employees trust that their organization prioritizes their well-being, they feel psychologically safe and empowered to speak up about safety concerns, which enables patient safety to improve in everyday clinical practice [33]. Empowered healthcare workers, in turn, have the ability to access resources, information, and support needed to perform work and gain opportunity to develop. They also tend to speak up about safety concerns or ethical conflicts, thereby enabling those issues to be escalated for swift resolution. Empowered workers are also resilient in implementing solutions to ensure patient safety, despite obstacles, and sharing those innovations with peers and managers, to enable learning and change [43,45]. According to the literature, empowerment can be achieved in the work environment through leader-empowering behaviors, including enhancing the meaningfulness of work; fostering opportunity to participate in decision making; facilitating the attainment of organizational goals; and providing autonomy from bureaucratic restrictions [44,45]. Therefore, worker trust, psychological safety, and empowerment are key elements of a positive work environment that enable safe, high-quality care to be delivered, despite challenges. In other words, they are pre-requisites for preserving organizational resilience, patient safety, and staff retention, during and beyond the pandemic.

### 4.2. Recommendation 2: Develop Communication Structures to Enable the Organization to Learn from the Problem-Solving Strategies and Communications of Individual Healthcare Workers

An environment that enables workers to communicate about safety concerns and workarounds needs to be combined with communication structures that enable the organization to learn from the individual-level communications and error recovery (problem-solving) strategies. This in turn, requires innovative communication structures that engage all levels of the organization, from frontline workers to senior leadership in communication related to patient safety, on an ongoing basis. An example of such a communication structure would be ‘tiered team huddles’ [50,51]. Tiered huddles are brief (approximately 15-min) meetings that cascade rapidly throughout the facility, beginning with the frontline level and progressing all the way to the senior leadership level, through the use of ‘boundary spanners,’ i.e., one or more representatives from each level (tier) in the next tier, to enable information from one tier’s huddles to be shared in the next. Tiered huddles enable open sharing of issues from the frontlines, with middle and senior leadership levels of the organization. As such, they have the potential to improve communication across boundaries to enable organizational learning, resilience, patient safety, and lasting improvement (change). A formal organizational performance improvement committee structure that utilizes ‘boundary spanners’ (as described above) also has the potential to achieve similar results [52,53].

Importantly, the use of boundary spanners in tiered huddles enables workers to adopt a learning mindset rather than a performance mindset. A learning mindset supports the generation of new ideas, while a performance mindset follows established protocols [54]. A learning culture, cultivated through innovative communication structures (like those described above), can enable workers to offer ideas for practice changes (e.g., bringing IV-bags to the hallway for drug titration versus the established practice of entering each patient’s room to perform titration) and to communicate those ideas to leadership to enable organizational learning [52,53,54,55]. Ideas for new practices in turn, could range from new roles (e.g., non-ICU nurse serving in ICU), new sites of care (e.g., use of makeshift ICUs), to new technologies (e.g., use of telehealth, social media, or digital patient navigation), which, when implemented consistently at the organizational level, could help to ensure patient safety and lasting improvement, even under challenging conditions [56].

It would be relevant to note, that the concept of Relational Coordination (RC) in the organization science literature serves to integrate both sets of recommendations (discussed above) into a cohesive whole. RC is a mutually reinforcing process of relating and communicating to enable task integration [57]. As such, RC has potential to transform relationships for high performance by developing shared goals, shared knowledge, and mutual trust and respect across boundaries, through communication that is accurate, timely, and problem-solving oriented. In other words, RC serves to integrate both sets of recommendations for HCO leaders discussed in this paper, i.e., (1) creating an environment of mutual trust to enable problem-solving communication to flow freely from individual healthcare workers to managers; and (2) developing a communication structure that enables the organization to learn from individual problem-solving strategies, to allow resilience to progress from reactive to proactive stages.

## 5. Conclusions

This paper applies the organizational resilience framework to discuss how a stoic approach to healthcare worker support during the COVID-19 pandemic has the potential to restrict organizational resilience, and adversely impact patient safety and staff retention during and beyond the pandemic period. To overcome these challenges, HCO leaders need to adopt a holistic consideration of worker psychological safety—one that recognizes the broader impact of emotional distress created by COVID-19. Meaningful support for emotional distress during the pandemic, enables healthcare workers to trust that their organization has their backs, which in turn, enables them to feel psychologically safe and empowered to communicate safety concerns and problem-solving strategies to managers, thereby enabling resilience to advance from individual to organizational levels. Concurrently, since learning from individual error recovery is crucial for advancing from reactive to proactive resilience at the organizational level, HCOs need to develop communication structures that enable the organization to learn from individual problem-solving strategies to prevent problem recurrence, reduce practice variation, increase patient safety, and ensure lasting improvement (change). Correspondingly, this paper puts forth two sets of recommendations for HCO leaders to preserve resilience, patient safety, and staff retention during and beyond the COVID-19 pandemic:

1. Create an environment of trust, psychological safety, and empowerment to enable individual workers to communicate patient safety concerns to managers.
2. Develop communication structures to enable the organization to learn from the problem-solving strategies and communications of individual healthcare workers.

As discussed in this paper, concurrent implementation of both sets of recommendations can enable HCO leaders to preserve organizational resilience, patient safety, and staff retention, to ensure sustainable HCO operations during and beyond the COVID-19 pandemic.
